# Maternal Obesity and Occurrence of Fetal Macrosomia: A Systematic Review and Meta-Analysis

**DOI:** 10.1155/2014/640291

**Published:** 2014-12-07

**Authors:** Laura Gaudet, Zachary M. Ferraro, Shi Wu Wen, Mark Walker

**Affiliations:** ^1^University of Ottawa, Faculty of Medicine, 451 Smyth Road, Ottawa, ON, Canada K1H 8M5; ^2^Division of Maternal-Fetal Medicine, Department of Obstetrics, Gynecology & Newborn Care, The Ottawa Hospital, 501 Smyth Road, Ottawa, ON, Canada K1H 8L6; ^3^Ottawa Hospital Research Institute, Ottawa, ON, Canada K1H 8L6; ^4^Healthy Active Living and Obesity (HALO) Research Group, Children's Hospital of Eastern Ontario, 401 Smyth Road, Ottawa, ON, Canada K1H 8L1

## Abstract

*Objective*. To determine a precise estimate for the contribution of maternal obesity to macrosomia. *Data Sources*. The search strategy included database searches in 2011 of PubMed, Medline (In-Process & Other Non-Indexed Citations and Ovid Medline, 1950–2011), and EMBASE Classic + EMBASE. Appropriate search terms were used for each database. Reference lists of retrieved articles and review articles were cross-referenced. *Methods of Study Selection*. All studies that examined the relationship between maternal obesity (BMI ≥30 kg/m^2^) (pregravid or at 1st prenatal visit) and fetal macrosomia (birth weight ≥4000 g, ≥4500 g, or ≥90th percentile) were considered for inclusion. *Tabulation, Integration, and Results*. Data regarding the outcomes of interest and study quality were independently extracted by two reviewers. Results from the meta-analysis showed that maternal obesity is associated with fetal overgrowth, defined as birth weight ≥ 4000 g (OR 2.17, 95% CI 1.92, 2.45), birth weight ≥4500 g (OR 2.77,95% CI 2.22, 3.45), and birth weight ≥90% ile for gestational age (OR 2.42, 95% CI 2.16, 2.72). *Conclusion*. Maternal obesity appears to play a significant role in the development of fetal overgrowth. There is a critical need for effective personal and public health initiatives designed to decrease prepregnancy weight and optimize gestational weight gain.

## 1. Introduction

The term macrosomia describes a newborn with an excessively high birth weight indicative of fetal overgrowth. Most studies define macrosomia as a birth weight greater than or equal to 4000 g; however others use 4500 g as the cut-point [1, 2]. There has been further interest in the group of infants whose birth weight exceeds 5000 g [3]. Based on the variation in cut-points, we propose that macrosomia can be subdivided into Class I (birth weight 4000–4499 g), Class II (4500–4999 g), and Class III (≥5000 g). Alternatively, fetal overgrowth can be defined as a birth weight greater than the 90th percentile, corrected for gestational age [4].

Excessive growth in the fetus is a major contributor to adverse obstetrical outcomes. Khashu et al. examined the perinatal outcomes of 1842 macrosomic newborns in British Columbia, and Canada and identified significantly increased maternal risks of emergency Caesarean section, obstetrical trauma, postpartum hemorrhage, and maternal diabetes (all outcomes, *P* < 0.001) [5]. Further, the infants were at higher risk of having birth trauma, of needing resuscitation, and of having an Apgar score less than seven at five minutes of life (*P* < 0.001) [5]. There is also evidence that macrosomia is associated with shoulder dystocia, brachial plexus injury, skeletal injuries, meconium aspiration, perinatal asphyxia, hypoglycemia, and fetal death [6]. Based on existing literature, there is little doubt that fetal macrosomia is associated with adverse pregnancy outcomes for both mother and infant. In addition, there is a recognized association between fetal macrosomia and long-term consequences for the newborn, including obesity, diabetes, and heart disease [7–20].

Although there is a plethora of information available in the literature regarding the contribution of maternal obesity, both preexisting and due to excessive gestational weight gain, to fetal macrosomia, the exact effect size of this relationship remains imprecise [4, 21–40]. At the time of our analysis, only one previous meta-analysis could be identified, in which the relationship between obesity and fetal overgrowth was examined as a secondary outcome [41]. Therefore, the objective of this project was to systematically review the literature regarding maternal obesity and fetal macrosomia and to complete a meta-analysis to provide the best possible estimate for the increase in macrosomia that can be attributed to maternal obesity.

## 2. Sources

The following databases were searched by a librarian experienced in systematic reviews: PubMed, Medline (In-Process & Other Non-Indexed Citations and Ovid Medline, 1950–2011), and EMBASE Classic + EMBASE. Databases were searched using a comprehensive and sensitive search strategy aimed at identifying as many studies as possible. The search strategy was formulated with the assistance of the librarians at the University of Ottawa. Results were filtered to include studies involving human subjects. The terms used in PubMed were as follows:

1. body mass index[mh] AND obesity[mh] AND (pregnancy complications[majr] OR pregnancy outcome[majr]),
2. ((inprocess[sb]) OR (publisher [sb])) AND (pregnan∗[Title] AND obes∗[Title]).

The terms used in Medline were as follows:

1. Exp Obesity/or obesity.mp,
2. Exp Body Mass Index/or BMI.mp,
3. 1 and 2,
4. Exp Pregnancy Complications or pregnancy complica∗.mp,
5. Exp Pregnancy Outcome/or pregnancy outcome∗.mp,
6. 3 or 4,
7. 3 and 6.

The terms used in EMBASE Classic + EMBASE were as follows:

1. exp MORBID OBESITY/or exp ABDOMINAL OBESITY/or exp OBESITY/or obesity.mp,
2. exp body mass/or body mass index.mp,
3. 1 and 2,
4. exp pregnancy complication/or pregnancy complic∗.mp,
5. exp pregnancy outcome/or pregnancy outcome∗.mp,
6. 3 or 4,
7. 3 and 6.

The references for the resulting studies were then reviewed to identify any additional studies that were not identified in the preliminary search. The full texts of articles that were felt to be potentially relevant were obtained. Finally, review articles on obesity and maternal outcomes published between 2000 and 2011 were reviewed and their reference lists searched for additional potential studies. We did not attempt to locate unpublished studies. Electronic messages were sent to some authors to obtain clarification where necessary.

## 3. Study Selection

Observational studies, including prospective and retrospective cohort studies as well as case-control studies were sought for inclusion. To be eligible for inclusion, studies had to identify cases using the Institute of Medicine (IOM) definition of obesity (BMI ≥30.0 kg/m^2^). Maternal obesity defined as prepregnancy, first trimester, or first antenatal visit BMI ≥30 kg/m^2^ comprised the exposure variable. There had to be sufficient data present to allow for quantification of the number of obese patients included in the study. Studies also had to identify a control group of women with a BMI in the underweight range (BMI <18.5 kg/m^2^), normal weight range (BMI 18.5–24.9 kg/m^2^), or combined underweight + normal weight range (BMI <25.0 kg/m^2^) that must have been obtained prepregnancy, in the first trimester, or at the first antenatal visit. Studies were included if maternal weight was obtained by self-report or direct measurement and infant birth weight was reported. For the outcome measures, studies had to include data that allowed for quantitative measurement of risk of overgrowth, defined as large for gestational age (≥90% ile) or fetal macrosomia (≥4000 g and/or ≥4500 g).

All studies with an English abstract were considered for inclusion. Studies that did not have full text in English were translated for review. All potential studies were assessed for eligibility by the first reviewer (LG) according to the prespecified criteria outlined in the previous sections. Studies and abstracts were screened and duplicates were removed. Data were extracted from each publication by the first reviewer. All identified studies were then reviewed by a second reviewer (ZF) and data extraction completed. Discrepancies regarding inclusion and extraction were then resolved by consensus.

The quality of included studies was assessed using criteria from the Newcastle-Ottawa Quality Assessment Scale [72]. The representativeness of the exposed and control groups, the means by which the exposure was ascertained, and follow-up rates were assessed. The overall quality of the included studies was then graded as low, moderate, or high according to prespecified criteria. All data were extracted independently by both reviewers and quality grades assigned; discrepancies were resolved by consensus.

A structured data form was developed prior to beginning data abstraction. Data from the different studies were then combined by meta-analysis. Frequencies were then used to generate unadjusted odds ratios and confidence intervals and Forest plots were generated. Meta-analysis was completed using the Comprehensive Meta-Analysis Version 2.0. A random effect model was used to estimate the overall effect [73]. To assess statistical heterogeneity and its magnitude, we used Cochran's *Q* (*α* = 0.10) and the *I* ^2^ statistic, respectively. A sensitivity analysis was then undertaken, including assessment of the effect of study quality.

## 4. Results

**Figure 1:**
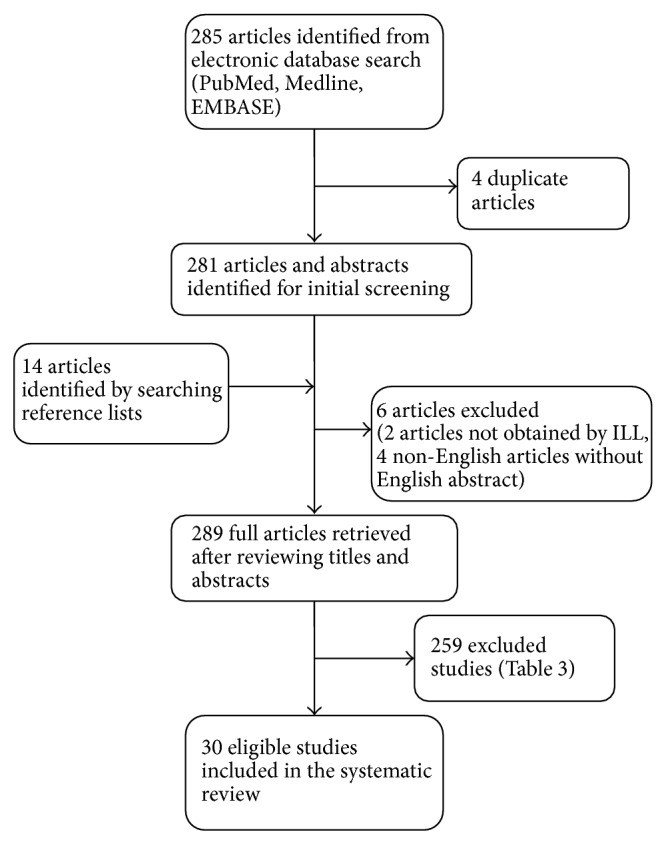
Study flow diagram.

Thirty studies met the inclusion criteria (Figure 1). The quality of studies was assessed for those included and excluded. Criteria for quality assessment were determined* a priori* (Table 1). Four studies were judged to be of high quality, fifteen were of moderate quality and eleven were of low quality. Quality assessment of the included studies [23, 24, 42–46, 48–59, 61–69, 71, 74] can be found in Table 2 and characteristics of excluded [4, 6, 21, 25, 27–29, 31, 34–39, 47, 60, 70, 75–307] studies can be found in Table 3. Of the included studies, nine were conducted in the United States, four in the United Kingdom, four in Denmark, two in Canada, two in Germany, and one in each of Hong Kong, Australia, Norway, Italy, India, France, Finland, Saudi Arabia, and the West Indies. Thus, the information in this review applies primarily to upper/middle income countries according to the World Bank classification [308]. The year of publication ranged from 1992 to 2010. Of included studies, eight had prospective cohort design, twenty-one had retrospective cohort design, and 1 was a retrospective case-control study. Eleven of the studies were conducted using population-based databases; these studies contributed 1,443,449 women to the meta-analysis.

**Figure 2:**
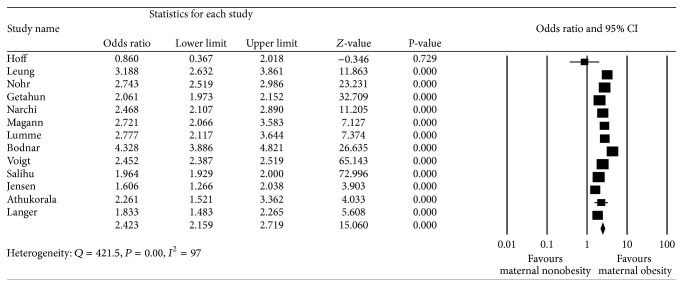
Forest plot for large for gestational age (>90% ile).

When studies were reviewed, the outcome measures of interest were identified. Six studies reported on more than one outcome measure; information for all relevant outcome measures was abstracted. Thus, thirteen studies reported on LGA, sixteen reported on macrosomia ≥4000 g, and eight reported on macrosomia ≥4500 g. In the thirteen studies that examined the relationship between maternal obesity and infant birth weight ≥90% ile, there were a total of 162,183 obese parturients. The control group consisted of 1,072,397 underweight or normal weight women. A total of 214,385 infants were large for gestational age (17.4%). Of these, 36,293 were born to obese mothers; thus, 22.4% of obese mothers gave birth to an LGA baby. By comparison, 16.6% of underweight or normal weight mothers gave birth to an LGA baby (*n* = 178,092). Meta-analysis revealed an overall unadjusted odds ratio of 2.42 (2.16,2.72) (Table 4, Figure 2).

**Figure 3:**
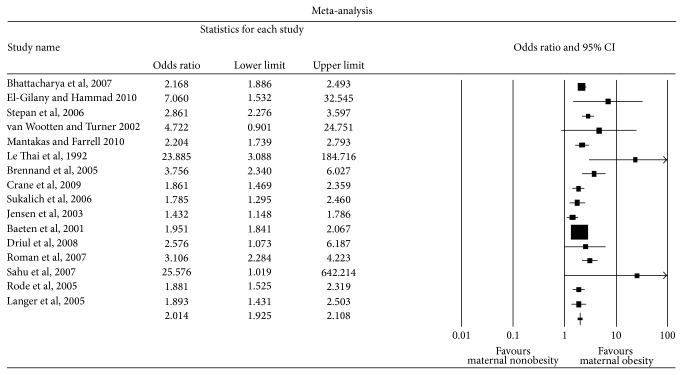
Forest plot for macrosomia (birth weight ≥4000 g).

In the sixteen studies that examined the relationship between maternal obesity and macrosomia ≥4000 g, there were a total of 20,693 obese parturients. The control group consisted of 110,696 underweight or normal weight women. A total of 13,612 infants had a birth weight ≥4000 g (10.4%). Of these, 3,275 were born to obese mothers; thus, 15.8% of obese mothers gave birth to a macrosomic baby weighing ≥4000 g. By comparison, 9.3% of underweight or normal weight mothers gave birth to a macrosomic baby weighing ≥4000 g (*n* = 10,337). Meta-analysis revealed an overall unadjusted odds ratio of 2.17 (1.92,2.45) (Table 3, Figure 3).

**Figure 4:**
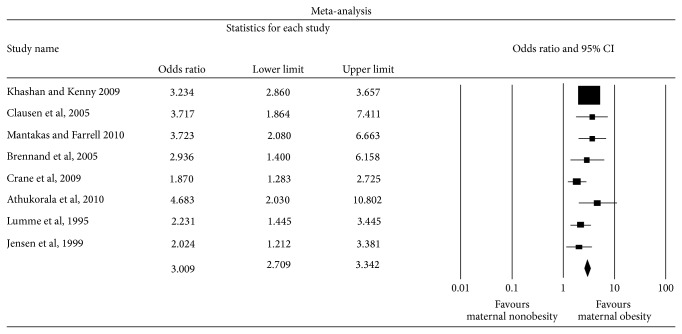
Forest plot for macrosomia (birth weight ≥4500 g).

In the eight studies that examined the relationship between maternal obesity and macrosomia ≥4500 g, there were a total of 18,909 obese parturients. The control group consisted of 62,712 underweight or normal weight women. A total of 1,739 infants had a birth weight ≥4500 g (2.1%). Of these, 746 were born to obese mothers; thus, 3.9% of obese mothers gave birth to an LGA baby. By comparison, 1.6% of underweight or normal weight mothers gave birth to an LGA baby (*n* = 993). Meta-analysis revealed an overall unadjusted odds ratio of 2.77 (2.22,3.45) (Table 3, Figure 4).

There was some important clinical heterogeneity between the included studies. For example, some studies included only normal weight patients in the control (17/30) while others included normal weight and underweight women (13/30). Also, most studies determined BMI using self-reported prepregnancy weight or did not provide information on how BMI was derived (20/30), while those studies that used measured weights had differing criteria for when that weight was measured (varied from <8 weeks to <16 weeks). Furthermore, some studies excluded women with hypertension or diabetes, while others included them.

There was also a marked amount of statistical heterogeneity, as assessed by the *I* ^2^ statistic. For obese women, the *I* ^2^ value for LGA was 97%, for macrosomia of ≥4000 g the *I* ^2^ value was 69%, and for macrosomia of ≥4500 g the *I* ^2^ value was 48%. These indicate diverse results and a large amount of heterogeneity that cannot be explained by chance alone. Sensitivity analysis showed that including only high quality studies decreased heterogeneity for LGA; the *I* ^2^ value improved to 0% from 97%. Including only high quality studies for LGA gives an odds ratio of 2.54 (95% CI 2.22, 2.92). As there was only one high quality study for macrosomia ≥4000 g, a similar analysis could not be undertaken. For macrosomia ≥4500 g, the *I* ^2^ value worsened slightly, from 48% to 62%.

## 5. Conclusion

This systematic review and meta-analysis confirms that maternal obesity is associated with fetal overgrowth. The odds of delivering an excessively large baby are increased: for large for gestational age infant (≥90th percentile) by 142%, for birth weight ≥4000 g by 117%, and for birth weight ≥4500 g by 277%. Determinants of macrosomia have been studied extensively. Identified risk factors include maternal prepregnancy diabetes (adjusted OR 4.6, 95% CI 2.57, 8.24), previous macrosomic birth (OR 3.1, 95% CI 2.61, 3.74), postterm pregnancy greater than 42 weeks gestation (OR 3.1, 95% CI 2.47, 3.86), maternal excess weight with BMI greater than 25 before pregnancy (OR 2.0, 95% CI 1.72, 2.32), male infant gender (OR 1.9, 95% CI 1.66, 2.21), gestational diabetes mellitus (OR 1.6, 95% CI 1.26, 2.16), and nonsmoking (OR 1.4, 95% CI 1.14, 1.82) [302]. Fetal growth is a complex biologic process that is regulated by both maternal and fetal factors including genes and environment. Maternal obesity likely contributes to macrosomia via mechanisms including increased insulin resistance (even in women who do not have diabetes) resulting in higher fetal glucose and insulin levels [309]. Placental lipases metabolize triglycerides in maternal blood, allowing free fatty acids to be transferred in excess to the growing fetus [310].

The sensitivity analysis suggested the importance of conducting well-designed high-quality studies. Of particular importance is ensuring that maternal weight and height are directly measured as early in pregnancy as possible. Data from a recent prospective cohort study found that pregnant women of all body masses under-report their prepregnancy weight when first trimester weight is used as a proxy which further substantiates the need for objective measurements [311]. The limitations of using either self-reported prepregnancy weight or first trimester weight as a surrogate for prepregnancy weight must be considered. Few women, however, will enter a different class of body mass on the basis of this potential misclassification bias.

The generalizability of the results should be interpreted with caution. The majority of the studies included in this review (including several national population-based cohorts) were completed in North America and Western Europe. Few studies examined the role of maternal obesity on fetal overgrowth in women from Africa, Asia, or South America. As there are fundamental differences in nutrition, socioeconomic and educational status, and prenatal/intrapartum care in these regions, results may or may not be applicable.

The results from this meta-analysis provide convincing evidence of the positive relationship between maternal obesity and fetal overgrowth. Clearly, optimization of weight prior to pregnancy is ideal; individual and public health measures should be in place to encourage women to have a normal body weight prior to pregnancy. Maternity and newborn care providers should be aware of the increased risk among obese women, encourage lifestyle modifications that decrease gestational weight gain, and manage abnormal glucose metabolism to optimize fetal growth. This is important to decrease both intrapartum complications and neonatal sequelae (such as birth trauma and hypoglycemia). Furthermore, optimal fetal growth contributes to* in utero* epigenetic programming that favours a healthy long-term weight trajectory and metabolic profile. The association between maternal obesity and fetal overgrowth may well represent the first opportunity through which obese mothers can modify the intergenerational obesity cycle and result in healthier, happier families.

## Figures and Tables

**Table 1:** Quality assessment criteria.

Quality assessment (QA) variable	Quality assessment criteria		
	Low	Moderate	High
Representativeness of exposed cohort	Selected group of users (e.g., nurses, volunteers)	Somewhat representative of the average obese pregnant woman in the community	Truly representative of the average obese pregnant woman in the community

Source of nonexposed cohort	Drawn from a different source than exposed cohort	N/A	Drawn from the same source as the exposed cohort

Ascertainment of exposure (obesity)	Self-report height and weight	Self-report height or weight	Measured height and weight

Comparability of cohorts	Comparable for less than 3 of the variables assessed	Comparable for 3 or 4 of the variables assessed	Comparable for at least 5 of the variables assessed

Adequacy of follow-up	Loss to follow-up rate >5% or no description of those lost	Subjects lost to follow-up unlikely to introduce bias (<5% loss to follow-up and description of those lost)	All subjects accounted for

Overall rating	Majority of QA variables rated as high, including ascertainment of exposure	Some QA variables rated as high, obesity self-reported	Few QA variables rated as high, obesity self-reported

**Table 2:** Quality assessment of included studies.

Study	Representativeness of the exposed cohort	Source of nonexposed cohort	Ascertainment of exposure (obesity)	Comparability of cohorts	Adequacy of follow-up	Overall rating
Hoff et al., 2009 [42]	**Moderate ** Outcome of second pregnancy in women who were overweight in their first pregnancy	**High ** Same population as exposed cohort	**Low ** No information	**Low ** Comparable for parity and race Not comparable for age and socioeconomic status No information on diabetes or hypertension	**High ** Retrospective cohort, 100% “follow-up”	**Low **

Salihu et al., 2009 [43]	**High** State-wide registry used to validate US national datasets	**High** Same population as exposed cohort	**Moderate** Self-reported prepregnancy weight, measured height	**Low** No comparable variables Not comparable for age, parity, diabetes, hypertension, or race No information on socioeconomic status	**High** Retrospective cohort, 100% “follow-up”	**Moderate**

Crane et al., 2009 [44]	**High** Provincial perinatal database	**High** Same population as exposed cohort	**Low** Self-reported prepregnancy weight and height	**Low** Comparable for age Not comparable for parity, diabetes, hypertension No information on socioeconomic status or race	**High** Prospective cohort, 100% “follow-up”	**Moderate**

Leung et al., 2008 [45]	**Low** Not enough information to determine	**High** Same population as exposed cohort	**Low** BMI obtained from weight and height at antenatal booking—unclear whether self-report or measured	**Low** Comparable for age and race Not comparable for parity, presence of diabetes, presence of hypertension No information on socioeconomic status	**High** Prospective cohort, 100% “follow-up”	**Low**

Nohr et al., 2008 [46, 47]	**High** Truly representative of the average obese pregnant woman in Denmark	**High** Same population as exposed cohort	**Low** Self-reported prepregnancy weight and height	**Low** Not comparable for age, parity, presence of diabetes, presence of hypertension, socioeconomic status No information on race	**Low** ~30% of women were excluded because they did not participate in the second interview, no description given	**Moderate**

Khashan and Kenny 2009 [48]	**High** Truly representative of the average obese pregnant woman in Manchester	**High** Same population as exposed cohort	**High** Measured height and first antenatal visit (around 16 weeks)	**Moderate** Comparable for age and socioeconomic status Not comparable for parity or race No information on presence of diabetes or hypertension	**High** Prospective cohort, 100% “follow-up”	**High**

Bhattacharya et al., 2007 [24]	**High** Truly representative of the average obese pregnant woman in Aberdeen and district	**High** Same population as exposed cohort	**High** Measured height and first antenatal visit (around 10 weeks)	**Low** Comparable for parity Not comparable for maternal age, presence of diabetes, presence of hypertension, socioeconomic status No information for race	**High** Prospective cohort, 100% “follow-up”	**High**

Getahun et al., 2007 [49]	**High** Truly representative of the average obese pregnant woman in Missouri	**High** Same population as exposed cohort	**Low** Self-reported prepregnancy weight and height	**Low** Not comparable for age, presence of diabetes, presence of hypertension or race No information for parity or socioeconomic status	**High** Retrospective cohort, 100% “follow-up”	**Moderate**

Sukalich et al., 2006 [50]	**Low** Selected group of users—<19 years old only	**High** Same population as exposed cohort	**Low** Self-reported prepregnancy weight and height	**Low** Comparable for presence of preexisting diabetes Not comparable for maternal age, parity, presence of hypertension, socioeconomic status, or race No information on multiple gestation	**High** Retrospective cohort, 100% “follow-up”	**Low**

Jensen et al., 2003 [51]	**Low** Selected group of users—women with a normal 75 g OGTT	**High** Same population as exposed cohort	**Low** No description of how prepregnancy BMI was obtained	**Low** Comparable for presence of diabetes Not comparable for age, parity, presence of hypertension, or race No information for socioeconomic status or multiple gestation	**High** Prospective cohort, 100% “follow-up”	**Low**

Stepan et al., 2006 [52]	**High** Truly representative of the average obese pregnant woman in Leipzig	**High** Same population as exposed cohort	**Low** No description of how prepregnancy BMI was obtained	**Low** Comparable for maternal age No information for parity, presence of diabetes, presence of hypertension, socioeconomic status, or race	**High** Retrospective cohort, 100% “follow-up”	**Low**

Athukorala et al., 2010 [53]	**Low** Selected group of users—women enrolled in the Australian Collaborative Trial of Supplements with antioxidants vitamin C and vitamin E	**High** Same population as exposed cohort	**High** Measured height and first antenatal visit	**Moderate** Comparable for age, parity, and race Not comparable for presence of diabetes, presence of hypertension, or socioeconomic status	Information not available	**High**

Narchi and Skinner 2010 [54]	**High** Truly representative of the average obese pregnant woman in the UK site	**High** Same population as exposed cohort	**High** Measured height and first antenatal visit (8–12 weeks)	**Low** Comparable for age Not comparable for parity, presence of diabetes, presence of hypertension, or race No information on socioeconomic status	**High** Retrospective cohort, 100% “follow-up”	**High**

Baeten et al., 2001 [23]	**High** Truly representative of the average obese pregnant woman in the state of Washington	**High** Same population as exposed cohort	**Low** Self-reported prepregnancy weight and height	**Low** Comparable for parity Not comparable for age, presence of diabetes, presence of hypertension, socioeconomic status, or race	**High** Retrospective cohort, 100% “follow-up”	**Moderate**

Clausen et al., 2005 [55]	**Low** Selected group of users (participants in a larger cohort study)	**High** Same population as exposed cohort	**Low** No description of how obesity was ascertained	**Low** No information given on age, parity, presence of diabetes, presence of hypertension, socioeconomic status, or race	**Low** Loss to follow-up 244/2294, 10.6%	**Low**

Driul et al., 2008 [56]	**High** Truly representative of the average obese pregnant woman in the state of Washington	**High** Same population as exposed cohort	**Low** Self-reported prepregnancy weight and height	**Low** No information given on age, parity, presence of diabetes, presence of hypertension, socioeconomic status, or race	**High** Retrospective cohort, 100% “follow-up”	**Low**

Roman et al., 2007 [57]	**High** Truly representative of the average obese pregnant woman on Reunion Island (consecutive cases)	**High** Controls derived from the same population as cases	**Low** No description of how obesity was ascertained	**Moderate** Comparable for age and parity Not comparable for presence of diabetes, presence of hypertension, or race No information on socioeconomic status	**High** Retrospectively derived cases and controls	**Moderate**

Sahu et al., 2007 [58]	**Moderate** Somewhat representative of the average obese woman in Northern India (had to deliver on site)	**High** Controls derived from the same population as cases	**Low** No description of how obesity was ascertained	**Moderate** Comparable for age and parity Not comparable for presence of diabetes or presence of hypertension No information on socioeconomic status or race	**High** Retrospectively derived cohort	**Low**

van Wootten and Turner 2002 [59]	**Low** Selected group—patients with gestational diabetes	**High** Controls derived from the same population as cases	**High** Measured height and first antenatal visit (8-9 weeks)	**Low** Comparable for presence of diabetes No information for age, parity, presence of hypertension, socioeconomic status, or race	**Low** 14 women were missing height and weight information	**Moderate**

Rode et al., 2005 [33, 60]	**High** Truly representative of the average obese pregnant woman in Copenhagen	**High** Controls derived from the same population as cases	**Low** Self-reported prepregnancy weight and height	**Low** Not comparable for presence of diabetes or presence of hypertension No information on age, parity, socioeconomic status, or race	**High** Retrospective cohort, 100% “follow-up”	**Moderate**

Magann et al., 2011 [61]	**Moderate** Somewhat representative of the average obese woman in Jackson or Portsmouth (two hospitals only, one naval)	**High** Controls derived from the same population as cases	**High** Measured height and first antenatal visit (all first trimester)	**Low** Not comparable for age, parity, presence of diabetes, presence of hypertension, or race No information for socioeconomic status	**High** Retrospective cohort, 100% “follow-up”	**Moderate**

Lumme et al., 1995 [62]	**High** Truly representative of the average obese pregnant woman in Northern Finland	**High** Controls derived from the same population as cases	**High** Measured height and first antenatal visit (all first visit)	**Low** Not comparable for age, parity, presence of diabetes, or presence of hypertension No information for socioeconomic status or race	**High** Prospective cohort, 100% “follow-up”	**High**

Langer et al., 2005 [63]	**Low** Selected group of users (women with GDM)	**High** Controls derived from the same population as cases	**Low** No description of how prepregnancy BMI was derived	**Low** Not comparable for age or parity No information for hypertension, socioeconomic status, race, or multiple gestation	**High** Prospective cohort, 100% “follow-up”	**Low**

Jensen et al., 1999 [64]	**Moderate** Somewhat representative of the average pregnant woman in Herning (several exclusion criteria)	**High** Controls derived from the same population as cases	**Low** No description of how obesity was ascertained	**Low** Comparable for presence of diabetes and presence of hypertension No information on age, parity, socioeconomic status, or race	**High** Retrospective cohort 100% “follow-up”	**Low**

Mantakas and Farrell 2010 [65]	**Low** Selected group of users (nulliparous women, one hospital site)	**High** Controls derived from the same population as cases	**Low** No description of how obesity was ascertained	**Low** Not comparable for age or race Comparable for parity No information for presence of diabetes, presence of hypertension, or socioeconomic status	**High** Retrospective cohort, 100% “follow-up”	**Low**

El-Gilany and Hammad 2010 [66]	**Low** Selected group of users—volunteers	**High** Same population as exposed cohort	**High** Measured height and first antenatal visit	**Low** Comparable for socioeconomic status Not comparable for age, parity, presence of diabetes, or presence of hypertension No information on race	**Moderate** Subjects lost to follow-up unlikely to introduce bias (<5% and description given)	**Moderate**

Bodnar et al., 2010 [67]	**High** Truly representative of the average obese pregnant woman in Pittsburgh, PA	**High** Same population as exposed cohort	**Low** Self-reported prepregnancy weight and height	**Low** Not comparable for age, parity, or race No information on presence of diabetes, presence of hypertension, or socioeconomic status	**High** Retrospective cohort, 100% “follow-up”	**Moderate**

Le Thai et al., 1992 [68]	**Moderate** Case definition adequate but not independently validated, consecutive cases	**High** Controls from same population as cases	**Low** Self-reported prepregnancy weight and height	**Low** Comparable for age Not comparable for parity, presence of diabetes, presence of hypertension No information for socioeconomic status or race	**High** Retrospective case control study, no loss to follow-up	**Moderate**

Voigt et al., 2008 [69, 70]	**High** Truly representative of the average obese pregnant woman in Germany	**High** Same population as exposed cohort	**High** Measured height and first antenatal visit	**Low** Comparable for age Not comparable for parity, presence of diabetes, or presence of hypertension No information on socioeconomic status or race	**High** Retrospective cohort, 100% “follow-up”	**High**

Brennand et al., 2005 [71]	**High** Truly representative of the average obese pregnant Cree woman in James Bay	**High** Same population as exposed cohort	**High** Measured height and first antenatal visit (<14 weeks)	**Low** Comparable for race Not comparable for age, presence of diabetes, or presence of hypertension No information on socioeconomic status or parity	**Low** 314 women were excluded because they did not have a recorded first weight <14 weeks (no description given)	**High**

**Table 3:** Characteristics of excluded studies.

Reason for exclusion	Number of studies excluded
Unrelated topic	62
Obesity not defined as BMI ≥30 kg/m^2^	83
Obesity measure not prepregnancy, first trimester, or first antenatal visit	5
Comparison group not one of BMI 18.5–24.9 kg/m^2^ or BMI <25.0 kg/m^2^	32
Data not present to allow quantitative analysis of obesity	15
Data not present to allow quantitative analysis of macrosomia	29
Meta-analysis	1
Review article	24
Comment	3
Case report	1
Duplicate articles	4
Total number excluded	**259**

**Table 4:** Association between maternal obesity and fetal overgrowth (odds ratios for individual studies and meta-analysis results).

Outcome of subgroup title	Study	Calculated unadjusted odds ratio	Reported adjusted odds ratio
Large for gestational age (≥90th percentile)	Hoff et al., 2009 [42]	0.86 (0.37, 2.02)	N/A
	Leung et al., 2008 [45]	3.19 (2.63, 3.87)	3.39 (2.78, 4.13)
	Nohr et al., 2008 [46, 47]	1.97 (1.81, 2.14)	N/A
	Getahun et al., 2007 [49]	2.06 (1.97, 2.15)	N/A
	Narchi and Skinner, 2010 [54]	2.47 (2.11, 2.89)	1.4 (1.3, 1.5)
	Magann et al., 2011 [61]	2.72 (2.07, 3.58)	3.10 (2.32, 4.15)
	Lumme et al., 1995 [62]	2.78 (2.12, 3.64)	2.3 (1.7, 3.0)
	Bodnar et al., 2010 [67]	4.33 (3.89, 4.82)	N/A
	Voigt et al., 2008 [69, 70]	2.54 (2.39, 2.52)	N/A
	Salihu et al., 2009 [43]	1.96 (1.93, 2.00)	N/A
	Jensen et al., 2003 [51]	1.61 (1.27, 2.04)	N/A
	Athukorala et al., 2010 [53]	2.26 (1.52, 3.36)	2.08 (1.47, 2.93)
	Langer et al., 2005 [63]	1.83 (1.48, 2.26)	N/A
	Total	**2.13 (2.10, 2.16)**	N/A

Macrosomia (birth weight ≥ 4000 g)	Bhattacharya et al., 2007 [24]	2.17 (1.89, 2.49)	N/A
	El-Gilany and Hammad, 2010 [66]	7.01 (1.52, 32.33)	N/A
	Stepan et al., 2006 [52]	2.86 (2.28, 3.60)	N/A
	van Wootten and Turner, 2002 [59]	4.72 (0.90, 24.75)	N/A
	Mantakas and Farrell, 2010 [65]	2.20 (1.74, 2.79)	1.9 (1.5, 2.5)
	Le Thai et al., 1992 [68]	23.88 (3.09, 184.72)	N/A
	Brennand et al., 2005 [71]	3.76 (2.34, 6.03)	3.73 (2.41, 5.05)
	Crane et al., 2009 [44]	1.86 (1.47, 2.36)	N/A
	Sukalich et al., 2006 [50]	1.78 (1.29, 2.46)	1.6 (1.2, 2.0)
	Jensen et al., 2003 [51]	1.43 (1.15, 1.79)	2.2 (1.6–3.1)
	Baeten et al., 2001 [23]	1.95 (1.84, 2.07)	2.1 (1.9, 2.3)
	Driul et al., 2008 [56]	2.58 (1.07, 6.19)	2.58 (1.08, 6.21)
	Roman et al., 2007 [57]	3.11 (2.28, 4.22)	3.1 (2.2, 4.3)
	Sahu et al., 2007 [58]	N/A	N/A
	Rode et al., 2005 [33, 60]	1.9 (1.53, 2.32)	1.8 (1.4–2.2)
	Langer et al., 2005 [63]	1.89 (1.43, 2.50)	N/A
	Total	**2.01 (1.93, 2.11)**	N/A

Macrosomia (birth weight ≥ 4500 g)	Khashan and Kenny, 2009 [48]	3.23 (2.86, 3.66)	2.71 (2.38, 3.07)
	Clausen et al., 2005 [55]	3.72 (1.86, 7.41)	4.3 (1.5, 12.1)
	Mantakas and Farrell, 2010 [65]	3.72 (2.08, 6.66)	8.7 (3.6–21.0)
	Brennand et al., 2005 [71]	2.94 (1.40, 6.16)	2.95 (1.87, 4.03)
	Crane et al., 2009 [44]	1.87 (1.28, 2.73)	N/A
	Athukorala et al., 2010 [53]	4.68 (2.03, 10.80)	4.54 (2.01, 10.24)
	Lumme et al., 1995 [62]	2.23 (1.45, 3.45)	1.8 (1.1, 2.8)
	Jensen et al., 1999 [64]	2.02 (1.21, 3.38)	N/A
	Total	**3.01 (2.71, 3.34)**	N/A
